# 3-Pyridyl Substituted Aliphatic Cycles as CYP11B2 Inhibitors: Aromaticity Abolishment of the Core Significantly Increased Selectivity over CYP1A2

**DOI:** 10.1371/journal.pone.0048048

**Published:** 2012-11-01

**Authors:** Lina Yin, Qingzhong Hu, Rolf W. Hartmann

**Affiliations:** 1 Pharmaceutical and Medicinal Chemistry, Saarland University & Helmholtz Institute for Pharmaceutical Research Saarland (HIPS), Saarbrücken, Germany; 2 ElexoPharm GmbH, Saarbrücken, Germany; Universidade Federal do Rio de Janeiro, Brazil

## Abstract

Aldosterone synthase (CYP11B2) is a promising therapeutic target for the treatment of cardiovascular diseases related to abnormally high aldosterone levels. On the basis of our previously identified lead compounds **I**–**III**, a series of 3-pyridinyl substituted aliphatic cycles were designed, synthesized and tested as CYP11B2 inhibitors. Aromaticity abolishment of the core was successfully applied to overcome the undesired CYP1A2 inhibition. This study resulted in a series of potent and selective CYP11B2 inhibitors, with compound **12** (IC_50_ = 21 nM, SF = 50) as the most promising one, which shows no inhibition toward CYP1A2 at 2 µM. The design conception demonstrated in this study can be helpful in the optimization of CYP inhibitor drugs regarding CYP1A2 selectivity.

## Introduction

Cardiovascular diseases are the leading cause of death in the United States and the majority of the European countries. It has been elucidated that some severe cardiovascular diseases such as hypertension, congestive heart failure (CHF) and myocardial fibrosis (MF) are closely associated with high aldosterone levels. [1] It is well known that aldosterone is a crucial hormone, which regulates electrolyte and volume homeostasis. After binding to mineralocorticoid receptors (MR), aldosterone promotes the retention of sodium and water at the expense of potassium excretion, subsequently resulting in the increase of blood volume and hypertension. Moreover, high aldosterone levels also stimulate synthesis and accumulation of collagens in cardiac fibroblasts leading to MF. The resulting increase in myocardial stiffness thereby causes diastolic dysfunction and ultimately heart failure [2].

**Figure 1:**
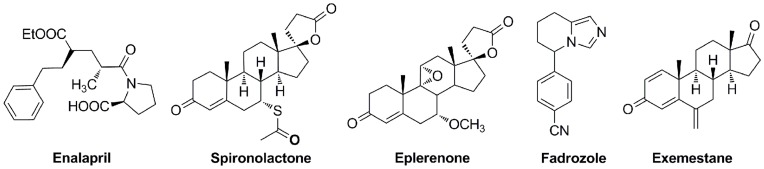
Structures of ACE inhibitor Enalapril, MR antagonists Spironolactone and Eplerenone, CYP11B2 inhibitor Fadrozole and aromatase inhibitor Exemestane.

**Figure 2:**
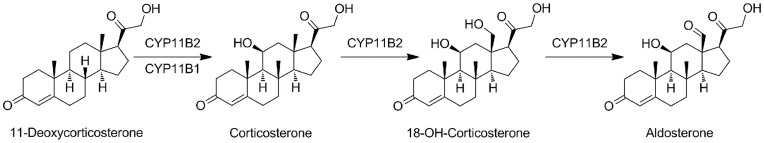
Biological synthesis of aldosterone catalyzed by CYP11B2.

Therefore, deprivation of aldosterone from its pathological effects is a feasible therapeutic approach to treat the related diseases. Currently, two main pharmacotherapies are clinically implemented to suppress the components of renin-angiotesin-aldosterone system (RAAS), which control the secretion of aldosterone via a negative feedback loop, including angiotensin-converting-enzyme (ACE) inhibitors such as enalapril and MR antagonists like spironolactone and eplerenone (Figure 1). ACE inhibitors are used for the treatment of hypertension and CHF by down-regulation of angiotensin II and subsequent aldosterone secretion. However, long-term suppressive effects of ACE inhibitors on plasma aldosterone levels are weakened due to the phenomenon known as “aldosterone escape”. [3] Although a clinical study revealed that blockade of MR by spironolactone has reduced the risk of both morbidity and mortality in patients with severe heart failure, the MR antagonists show severe adverse effects such as gynaecomastia or breast pain due to their steroidal structure exhibiting residual affinity to other steroid receptors. [4] Despite the fact that eplerenone as a selective MR antagonist achieves some improvement in terms of side effects as compared to spironolactone, severe hyperkalemia and weaker potency have been reported. [5] Furthermore, treatment with blockade of MR leaves high levels of aldosterone unaffected, which can result in further exacerbation of heart function in a MR independent nongenomic manner. [6] CYP11B2 is a mitochondrial cytochrome P450 enzyme catalyzing the conversion of 11-deoxycorticosterone to aldosterone in three consecutive steps (Figure 2). [7] Its inhibition was proposed as a new strategy for the treatment of aldosterone related cardiovascular diseases as early as 1994. [8] Recent *in vivo* studies in rats have demonstrated that CYP11B2 inhibitors can reduce plasma aldosterone levels. [9] Long-term administration of FAD286 (R-enantiomer of fadrozole, Figure 1) to rats with heart failure improves cardiac haemodynamics and cardiac function, which is more significant than those by spironoloactone. [10] However, FAD286 also shows strong inhibition of CYP11B1 and CYP19, thus urging us to design selective CYP11B2 inhibitors.

Our group has designed and synthesized several series of CYP11B2 inhibitors. [11]–[16] These compounds not only exhibited potent inhibition toward CYP11B2, but also showed good selectivity over CYP11B1, which is the key enzyme involved in glucocorticoid biosynthesis. This selectivity is very difficult to achieve due to the high homology up to 93% between these enzymes. However, some of these potent compounds showed strong inhibition of CYP1A2, which is probably due to the planar aromatic structure of the molecules. Therefore, in this study the aromaticity abolishment of the core was performed to reduce the CYP1A2 inhibition leading to a series of 3-pyridinyl substituted aliphatic cycles **1**–**21**. The percent inhibition and IC_50_ values of the synthetic compounds for CYP11B2 and CYP11B1 are presented in comparison to fadrozole. Inhibition of CYP1A2 was only tested for potent and selective compounds **2**, **4**, **7**, **8** and **10**.

### Design of Inhibitors

**Figure 3:**
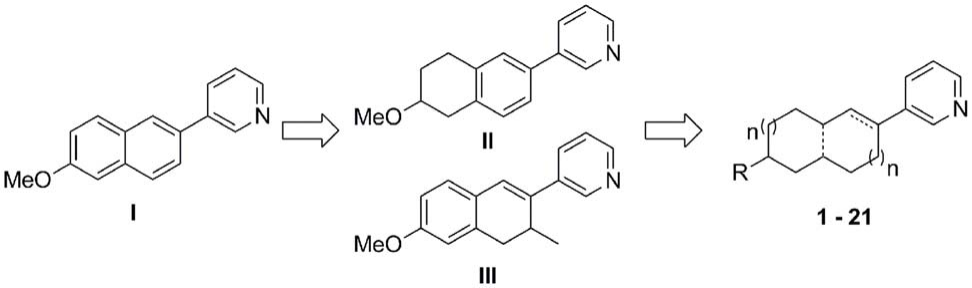
Conception of inhibitors design.

In the last decade, a wide range of compounds were designed as CYP11B2 inhibitors [17]–[19] based on the mechanism that a *sp^2^* hybrid N of the inhibitors could coordinate to the heme iron located in the center of protoporphyrin ring. This mechanism was primarily identified for aromatase inhibitors, [20]–[27] but was soon proven to be valid for inhibitors of other steroidogenic enzymes such as CYP11B1 [28]–[30] and CYP17 [31]–[42]. Since all cytochrome P450 enzymes, not only steroidogenic but also hepatic CYPs, consist of a heme moiety as the catalyzing unit, they are potential targets for inhibitors acting by this mechanism. Therefore, it is crucial to develop CYP11B2 inhibitors exhibiting selectivity over the other enzymes, especially CYP11B1. Another focus of selectivity is hepatic CYP enzymes due to their important roles in the metabolism of drugs and xenobiotics to prevent toxic effects. The inhibitors previously identified in our group have been demonstrated to be quite potent toward CYP11B2 and selective over CYP11B1. However, regarding CYP1A2, which is responsible for metabolizing neutral or basic planar substances, the selectivity needs further improvement. [43] Since the heterocycles providing the *sp^2^* hybrid N are always similar, the key to selectivity lies in the hydrophobic core. It can be seen that compound **I** [11] (Figure 3) with a naphthalene core showed strong CYP1A2 inhibition of 98% at a concentration of 2 µM. Saturation of the left cycle leading to tetrahydronaphthalene slightly reduced the CYP1A2 inhibition of the resulting compound **II** [15] (Figure 3) to 80%. Moreover, semi-saturation of the right cycle gently increased selectivity over CYP1A2 with 73% inhibition (compound **III**, [17] Figure 3). Concurring with this improvement, it is important that the high inhibitory potency toward CYP11B2 and good selectivity against CYP11B1 were sustained. After comparison of these lead compounds, it is apparent that abolishing the aromaticity of the core to impair the planarity is a feasible way for CYP11B2 inhibitors to increase their selectivity over CYP1A2. Based on this hypothesis, further reduction of aromaticity was pursued by saturating both cycles of the core. Moreover, the influences of different cycle size, the presence of H-bond forming groups and the removal of bridge bond were also investigated. Since these modifications resulted in highly flexible molecules, some rigid non-aromatic cores such as 3-bicyclo[2.2.1]heptane and 8-aza-bicyclo[3.2.1]octane were also employed.

## Results and Discussion

### Chemistry

**Figure 4:**
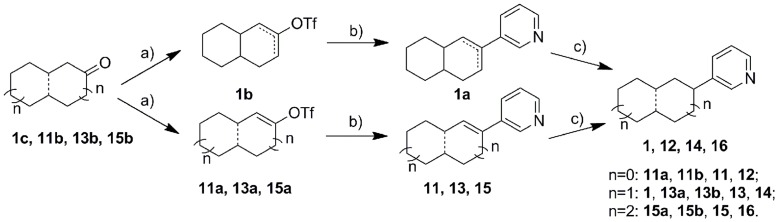
Reagents and conditions. a) Method A: Tf_2_O, CH_2_Cl_2_, 2,6-di-tert-butyl-4-methylpyridine, 2 h; b) Method B: Pd(PPh_3_)_4_, pyridine-3-boronic acid, Na_2_CO_3_, DME, H_2_O, 90°C, 2 h; c) Method C: 5% Pd/C, MeOH, H_2_, RT, 2 d.

**Figure 5:**
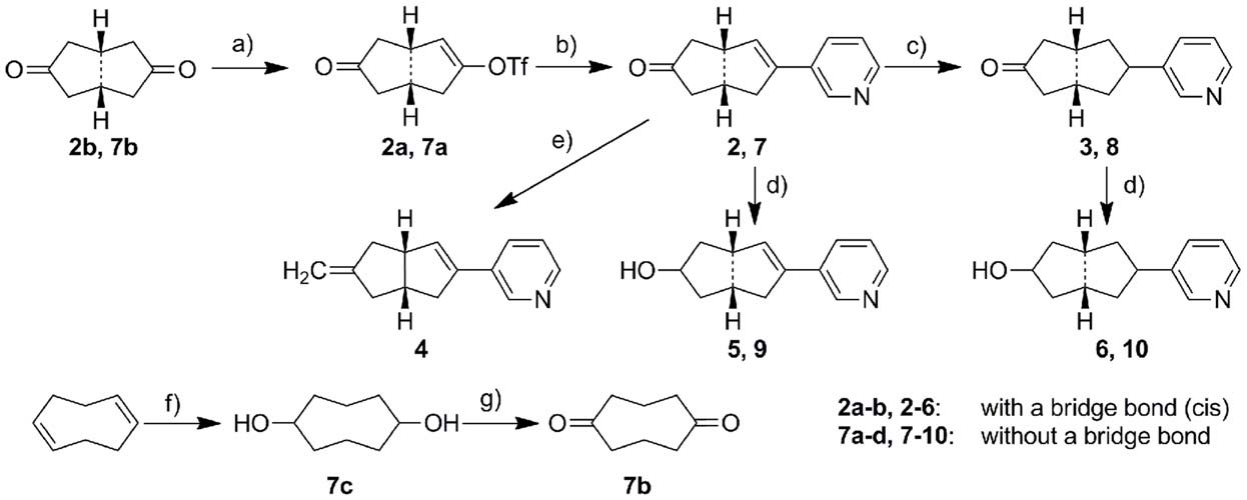
Reagents and conditions. a) Method A: Tf_2_O, CH_2_Cl_2_, 2,6-di-tert-butyl-4-methylpyridine, 2 h; b) Method B: Pd(PPh_3_)_4_, pyridine-3-boronic acid, Na_2_CO_3_, DME, H_2_O, 90°C, 2 h; c) Method C: 5% Pd/C, MeOH, H_2_, RT, 2 d. d) Method D: NaBH_4_, MeOH, RT, 2 h; e) CH_3_PPh_3_Br, n-BuLi, THF, 30 min at −78°C, 2 h at RT, f) BH_3_-THF, 3M NaOH, 35% H_2_O_2_, THF, g) PCC, CH2Cl2, reflux 2 d.

**Figure 6:**
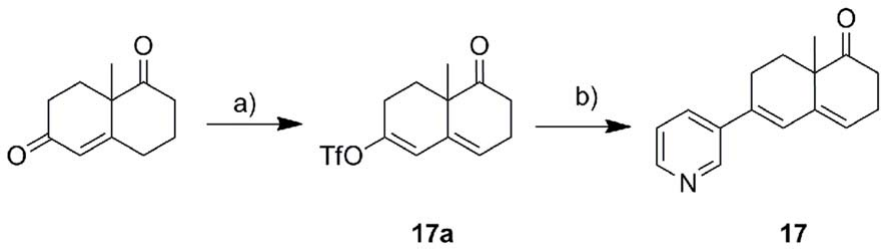
Reagents and conditions. a) Method A: Tf_2_O, CH_2_Cl_2_, 2,6-di-tert-butyl-4-methylpyridine, 2 h; b) Method B: Pd(PPh_3_)_4_, pyridine-3-boronic acid, Na_2_CO_3_, DME, H_2_O, 90°C, 2 h.

**Figure 7:**
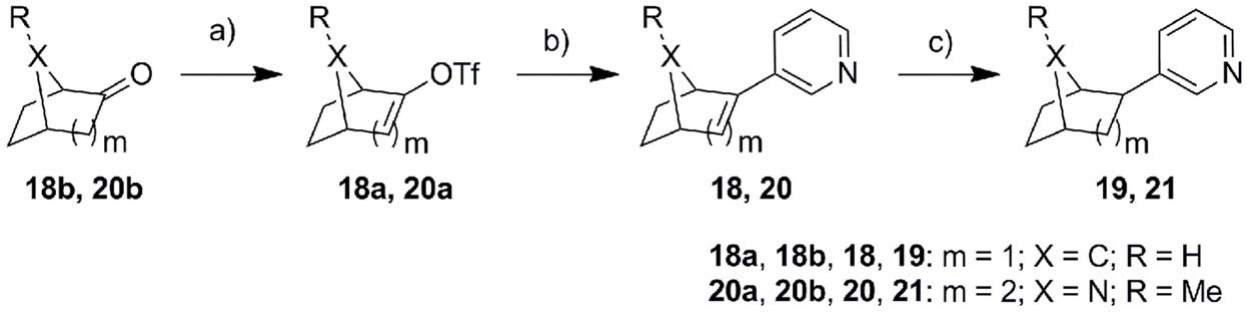
Reagents and conditions. a) Method A: Tf_2_O, CH_2_Cl_2_, 2,6-di-tert-butyl-4-methylpyridine, 2 h; b) Method B: Pd(PPh_3_)_4_, pyridine-3-boronic acid, Na_2_CO_3_, DME, H_2_O, 90°C, 2 h; c) Method C: 5% Pd/C, MeOH, H_2_, RT, 2 d.

The synthetic strategy employed for all final products is shown in Figures 4, 5, 6, and 7. The triflation of the starting ketones using trifluoromethanesulfonic anhydride and 2,6-di-*tert*-butyl-4-metyhylpyridine gave the corresponding enol triflates. These triflates were then coupled with 3-pyridyl boronic acid leading to the corresponding products with an α-double bond (**2**, **7**, **11**, **13**, **15**, **18** and **20**). The subsequent hydrogenation catalyzed by Pd/C was used to reduce the α-double bond and thereby to yield the saturated compounds (**1**, **3**, **8**, **12**, **14**, **16**, **19** and **21**). Several interesting points need to be addressed regarding the triflation. Firstly, **1b** obtained from the triflation of octahydronaphthalen-2(1*H*)-one was a mixture of two isomers with *Δ* 1, 2- or *Δ* 2, 3- double bond probably as the respective thermodynamic and kinetic products. Secondly, one equivalent of trifluoromethanesulfonic anhydride needs to be strictly controlled in the triflation of diones **2b** and **7b**, which were converted to the respective ketone products **2**, **3**, **7** and **8** following the general strategy described above. These ketone analogues were then reduced to hydroxyl compounds **5**, **6**, **9** and **10** using NaBH_4,_ whereas compound **3** underwent Wittig reaction to provide the methylidene compound **4**. Finally, as for the asymmetric (±) 9-methyl-5(10)-octaline-1,6-dione, the triflation occurred selectively in 6-position with the rearrangement of conjugative double bonds resulting in **17a**, which after Suzuki coupling led to **17**. Furthermore, compound **21** was obtained as an endo/exo mixture in a ratio of 5∶1, which was tested without further separation.

### CYP11B1 and CYP11B2 Inhibition

**Figure 8:**
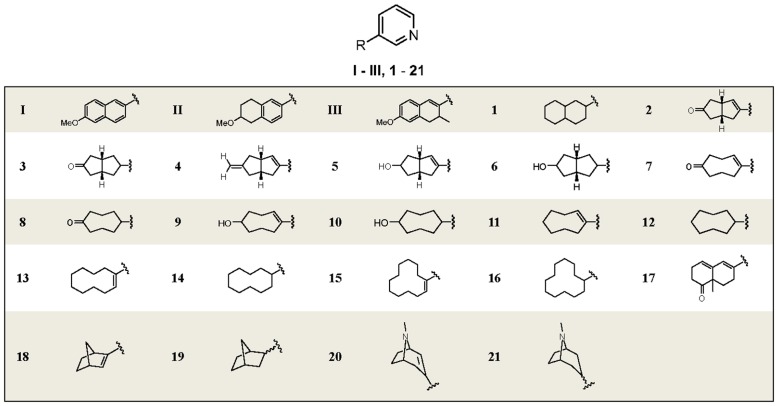
Structures of references I–III and compounds 1–21.

The synthesized compounds were tested for their inhibitory potencies against CYP11B2 [44] and CYP11B1 [45] with V79 MZh cells expressing the respective enzymes. The results are presented in Table 1 (for detailed structures, see Figure 8) with fadrozole as a reference, which showed strong inhibition against these two enzymes with IC_50_ values of 0.8 and 6.3 nM of CYP11B2 and CYP11B1, respectively.

**Table 1:** Inhibition of human CYP11B1 and CYP11B2 by references **I**–**III** and compounds **1**–**21**.

	11B2 IC_50_ (nM)[a]	11B1 IC_50_ (nM)[a]	SF[b]		11B2 IC_50_ (nM)[a]	11B1 IC_50_ (nM)[a]	SF[b]
**I**	6±2	1577±97	263	**II**	3±1	1145±78	347
**III**	3±1	248±19	79	**1**	157±14	611±34	4
**2**	34±5	282±17	8	**3**	258±22	306±25	1
**4**	6±2	205±12	34	**5**	225±21	663±27	3
**6**	347±35	133±29	0.4	**7**	205±16	851±58	4
**8**	443±36	1352±79	3	**9**	141±11	348±25	2
**10**	294±23	246±36	0.8	**11**	81±7	195±15	2
**12**	21±5	1047±67	50	**13**	21±3	258±16	12
**14**	22±2	872±79	40	**15**	1167±97	>10000	nd[c]
**16**	636±32	1772±56	3	**17**	462±36	1015±78	2
**18**	889±57	2132±113	3	**19**	99±21	137±12	0.9
**20**	2442±88	>5000	nd[c]	**21**	2835±135	>10000	nd[c]
**Fad**	0.8±0.05	6.3±0.6	8				

[a] Hamster fibroblasts expressing human CYP11B1 or CYP11B2, respectively; substrate: deoxycorticosterone, 100 nM.

[b] SF: selectivity factor = IC_50 CYP11B1_/IC_50 CYP11B2_.

[c] Fad: fadrozole; nd: not determined.

Intermediate **1a**, as a mixture of isomers with the double bond at different positions, exhibited 96% inhibition of CYP11B2 at 500 nM. This mixture also showed selectivity toward CYP11B1 with around 50% inhibition at the same concentration. However, due to the unsuccessful separation of these isomers, intermediate **1a** was finally abandoned. After hydrogenation of compound **1a**, the resulting saturated analogue **1**, however, showed a decreased inhibitory potency of 157 nM against CYP11B2, but similar inhibition of CYP11B1 (IC_50_ = 611 nM). This result indicates the importance of the double bond for inhibitory potency and selectivity. To further investigate this observation, the ring size was reduced to avoid double bond isomers, and a series of octahydropentalene analogues furnished with oxo, hydroxy or methylidene were synthesized. The double bond analogue **2** consisting of an oxo group exhibited an IC_50_ value of 34 nM and a selectivity factor of 8, whereas the corresponding saturated compound **3** was weaker (IC_50_ = 258 nM) and showed no selectivity. A similar result was observed for the hydroxy analogues **5** and **6**. The saturated hydroxy compound **6** even showed preference for CYP11B1 (IC_50_ of 133 nM for CYP11B1 *vs* 347 nM for CYP11B2). Moreover, since an unsubstituted methylidene can covalently bind to CYP enzymes leading to an irreversible binding as observed for the aromatase inhibitor exemestane (Figure 1) in clinical use for the treatment of breast cancer, a methylidene group was introduced into the molecule. As expected, compound **4** turned out to be the most potent inhibitor in this study with an IC_50_ value of 6 nM and a selectivity factor of 34. The good selectivity achieved over CYP11B1 indicates this methylidene only specifically binds to the corresponding amino acid residues present inside CYP11B2 but not CYP11B1.

Furthermore, to achieve more flexibility, the bridge bond was removed, resulting in a series of aliphatic cycles (compounds **7**–**16**). When substituted by similar hydrogen bond forming groups such as oxo or hydroxy, the resulting compounds exhibited variation of activity profiles compared to the corresponding bridged compounds. For the ketone analogues **7** (IC_50_ = 205 nM) and **8** (IC_50_ = 347 nM), the activities were slightly reduced compared to the corresponding compound **2** and **3** (IC_50_ = 34 and 258 nM, respectively), while the selectivities were maintained or were even better. Hydroxy analogue with a double bond (compound **9**) showed an increased activity (IC_50_ = 141 nM) against CYP11B2, but the inhibition of CYP11B1 also increased accordingly to 348 nM, leading to a reduction of selectivity (SF = 2). On the contrary, the saturated compound **10** exhibited similar inhibitory potency (IC_50_ = 294 nM) toward CYP11B2 but no selectivity.

Moreover, the influence of ring size on the inhibitory potency and selectivity was also investigated. The cyclodecane analogues, compounds **13** and **14** showed inhibitory potency with IC_50_ values around 20 nM, regardless of the presence of the double bond. Nevertheless, double bond analogue **13** was less selective compared to the saturated compound **14** (selectivity factor of 12 *vs.* 40 for compounds **13** and **14**, respectively). After the ring size was reduced from cyclodecane to cyclooctane, the activities were slightly increased. The saturated compound **12** turned out to be very potent (IC_50_ = 21 nM), and this compound was also the most selective inhibitor throughout this study (SF = 50). However, the increase of the ring size to cyclododecane was not tolerated. The inhibitory activities of the resulting compounds **15** and **16** were largely reduced to more than 500 nM.

It is notable that for compounds furnished with a hydrogen bond forming group like ketone or hydroxyl (compounds **2**, **3**, **5**–**10**), the analogues with a double bond are always more potent and selective than the corresponding saturated analogues regardless of the presence of the bridge bond. On the contrary, double bond renders minor difference on the CYP11B2 inhibition for compounds without hydrogen bond forming groups (compounds **11**–**16**). This observation is most likely a consequence of different orientations of the compounds in the enzyme active site, which are probably caused by some interactions between hydrogen bond forming groups and certain polar amino acid residues. Moreover, comparing among the cyclooctane derivatives **7**–**12**, it can be found that the introduction of hydrogen bond forming groups (ketone or hydroxyl) always decreased inhibitory potency toward CYP11B2.

With the intention of mimicking the natural substrate of CYP11B2, the unsaturated decalone analogue **17** was synthesized. However, only modest inhibition (IC_50_ = 462 nM) was observed.

On the other hand, the attempt to rigidify the core structure with a one-atom bridge (C or N) resulted in compounds **18**–**21**. When the bicyclo[2.2.1]heptane core was bearing the double bond (compound **18**) only modest inhibition toward CYP11B2 (IC_50_ = 889 nM) was observed. Per contra, the saturated compound **19** was more potent (IC_50_ = 99 nM), but not selective. However, for the [3.2.1] aza-bicycle analogues (**20** and **21**), a severe loss of activity (IC_50_>2000 nM) was observed probably as a consequence of the un-tolerable bulky bicycle core.

### CYP1A2 Inhibition

With the aim of overcoming a common disadvantage of naphthalene type or partially saturated naphthalene type CYP11B2 inhibitors – high CYP1A2 inhibition – this series of 3-pyridinyl substituted aliphatic cycles, which are completely non-aromatic, were synthesized. Seven very potent (IC_50_<100 nM) CYP11B2 inhibitors **2**, **4**, **11**–**14** and **19** were selected to determine CYP1A2 inhibition (Table 2). As expected, all compounds exhibited largely reduced inhibition of CYP1A2 compared to lead compounds **I**–**III** (IC_50_ of 41, 440 and 741 nM, respectively). It is striking that compounds **2** and **11**–**14** showed IC_50_ values more than 100 µM and 44 µM was observed for compound **19**. In contrast to no CYP1A2 inhibition by compound **2** with oxo, the methylidene analogue **4** showed moderate inhibition of 2171 nM, which is still much better than reference compounds **I**–**III**. A probable explanation for this could be that the methylidene group is oxidized by CYP1A2, and the resulting epoxide binds to the protein covalently. From these results it is apparent that aromaticity abolishment of the lipophilic core strongly decreased inhibition of hepatic CYP1A2.

**Table 2:** Inhibition of human hepatic enzyme CYP1A2 by selected compounds.

Compd	IC_50_ CYP1A2 nM [a]	Compd	IC_50_ CYP1A2 nM [a]
**I**	41±5	**11**	>100000
**II**	440±11	**12**	>100000
**III**	741±55	**13**	>100000
**2**	>100000	**14**	>100000
**4**	2171±97	**19**	44425±388

[a] Recombinantly expressed enzyme from baculovirus infected insect microsomes (Supersomes); furafylline: IC_50_ = 2420 nM.

### Docking Study

**Figure 9:**
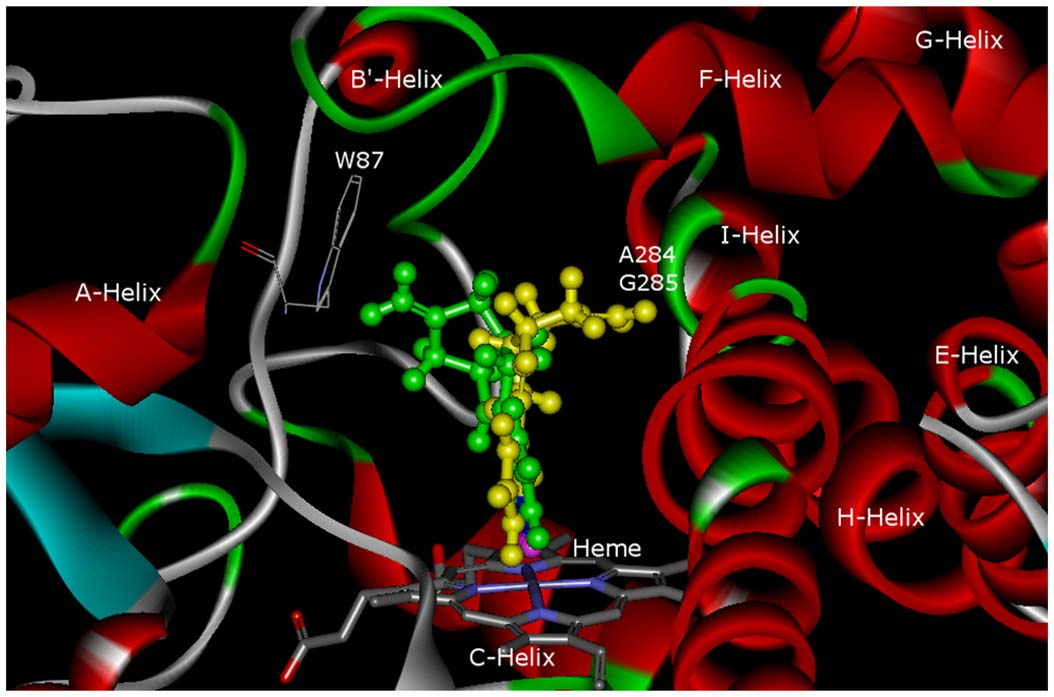
Binding of compound 4 into CYP11B2 homology model.

To elucidate the binding to the enzyme pocket, the most potent inhibitor (compound **4**) was docked into the human CYP11B2 homology model [18]. Two binding modes were observed differing only in the orientation of methylidene and the fusing cyclopentyl part of hexahydropentalene (Figure 9). As expected, the pyridyl coordinated to heme iron with its *Sp^2^* hybrid N in a perpendicular manner. The body of molecule paralleled I-helix indicating the π-π interactions between the double bond in hexahydropentalene and π-system of the amino acid backbone in the I-helix (Asp288, Thr289 and Thr290). In one binding mode, the fusing cyclopentyl part of hexahydropentalene and the attached methylidene oriented toward I-helix (backbones of Ala284 and Gly285). On the contrary, in the other binding mode, these moieties turned into the opposite direction and pointed to Trp87. It is notable that this orientation of methylidene is similar to the situation of exemestane in CYP19 crystal (PDB ID: 3S7S), where the methylidene located adjacently to Trp180. Since methylidene is reactive, it can probably covalently bind to the amino acid residues mentioned above and contribute to strong inhibition.

### Conclusion

In this study, 21 analogues of 3-pyridinyl substituted aliphatic cycles were designed, synthesized and evaluated as CYP11B2 inhibitors. Although the design conception focused on the improvement of CYP1A2 selectivity, these compounds exhibited potent inhibition of CYP11B2 and good selectivity over CYP11B1. Compounds **2**, **4**, **12**, **13** and **14** showed IC_50_ values ranging from 6 to 34 nM, with compound **4** being the most potent one (IC_50_ = 6 nM) and compound **12** being the most selective one (SF = 50). It has been observed that an α-double bond in analogues with a bridge bond or H-bond forming groups furnishing aliphatic cycles promoted inhibitory activity and selectivity, whereas deterioration was observed for 3-pyridinyl substituted alphatic cycles without H-bond forming groups. Surprisingly, with regard to the other compounds, the presence of H-bond forming groups reduced inhibitory potency probably due to a change in the binding mode. After removal of the bridge bond, the more flexible compounds did not show stronger inhibition. Moreover, the introduction of a methylidene as a potential reactive center increased not only CYP11B2 inhibition, but also the undesired CYP1A2 inhibition. The flexible 8 to 10 membered or bicyclic rings are appropriate, whereas flexible 12-membered ring or rigid bicyclic analogues are too bulky to be tolerated.

Furthermore, the design conception employed in this study, i.e. aromaticity abolishment of the lipophilic core and reduction of planarity to reduce CYP1A2 inhibition, was proven successful. For the five most potent and selective CYP11B2 inhibitors in this study, the CYP1A2 inhibition was determined and found to be largely reduced compared to their aromatic precursors. Among them, compounds **2**, **12** and **13** showed no inhibition at 2 µM. This is very important, since selectivity against hepatic CYP enzymes is a key issue for safety in drug discovery. Especially in the CYP enzyme inhibitors field, it is difficult to develop compounds without inhibition of crucial CYP1A2. The design conception demonstrated in this study could be helpful in the optimization aiming at the improvement of CYP1A2 selectivity.

## Methods

### Inhibition of CYP11B1 and CYP11B2

V79MZh cells expressing human CYP11B1 [45] or CYP11B2 [44] were incubated with [^14^C]-11-deoxycorticosterone as substrate. The assay was performed as previously described [44].

### Inhibition of Human Hepatic CYP1A2

The recombinantly expressed CYP1A2 enzyme from baculovirus-infected insect microsomes (commercially obtained from BD Gentest) was used and the assay was performed according to the manufacturer’s instructions (www.gentest.com).

### Chemistry

Melting points were determined on a Mettler FP1 melting point apparatus and are uncorrected. IR spectra were recorded neat on a Bruker Vector 33FT-infrared spectrometer. ^1^H-NMR and ^13^C-NMR spectra were measured on a Bruker DRX-500 (500 MHz). Chemical shifts are given in parts per million (ppm), and TMS was used as an internal standard for spectra obtained in CDCl_3_. All coupling constants (*J*) are given in Hz. ESI (electrospray ionization) mass spectra were determined on a TSQ quantum (Thermo Electron Corporation) instrument. High-resolution mass spectra were measured using an LTQ Orbitrap (Thermo Electron Corporation) with positive ESI. The purities of the final compounds were controlled by Surveyor®-LC-system. Purities were greater than 98%. Column chromatography was performed using silica-gel 60 (50–200 µm), and reaction progress was determined by TLC analysis on Alugram® SIL G/UV_254_ (Macherey-Nagel). Ketones and precursors of ketones used as starting materials were commercially obtained.

### Method A: Triflation

To a solution of 2,6-di-*tert*-butyl-4-methylpyridine (7.23 mmol) and the corresponding ketone (6.57 mmol) in CH_2_Cl_2_ (20 mL) was added trifluoromethanesulfonic anhydride (7.16 mmol) dropwise at 0°C under nitrogen. A white precipitate was formed thereafter. The reaction mixture was warmed to room temperature and further stirred for 4 h. The solvent was removed in vacuo, and the resulting residue was diluted with petroleum ether (20 mL). After removal of the solid by filtration, the filtrate was concentrated *in vacuo* to give a crude product which was used in the next step without further purification.

### Method B: Suzuki Coupling

A suspension of enol triflate (4.56 mmol), pyridine-3-boronic acid (5.92 mmol), sodium carbonate (22.8 mmol) and tetrakis(triphenylphosphine)palladium (0) (0.23 mmol) in dimethoxyethanol (45 mL) and water (15 mL) was stirred at 90°C under nitrogen for 2 h. The reaction mixture was cooled to room temperature slowly and diluted with water (10 mL). The aqueous layer was extracted with ethyl acetate (2×30 mL) and the combined organic layers were washed with brine twice and dried over MgSO_4_. After evaporation *in vacuo*, the resulting residue was purified by flash chromatography to afford the corresponding product.

### Method C: Hydrogenation

A mixture of 3-pyridyl derivative with an α-double bond (0.50 mmol) and 5% Pd/C (45 mg) in methanol (15 mL) was flushed with hydrogen for 2 d at room temperature The mixture was filtered through a Celite cake and concentrated *in vacuo* to afford the corresponding product.

### Method D: Sodium Borohydride Reduction

To a solution of a ketone (0.25 mmol) in methanol (5 mL) was added sodium borohydride (0.50 mmol) at room temperature. The reaction was stirred for 4 h. After removal of MeOH in vacuo, the residue was eluted with water (5 mL) and then extracted with ethyl acetate (3 × 5 mL). The combined organic layers were washed with brine, dried over MgSO_4_ and concentrated *in vacuo*. The residue oil was purified by flash chromatography to give the title alcohol.

#### 3-(Decahydro-naphthalen-2-yl)-pyridine (1)

The title compound was synthesized according to Method C using **1a** (300 mg, 1.41 mmol) and 5% Pd/C (45 mg) in methanol (15 mL) to give a pale yellow oil (257 mg, 85%): *R*_f_ = 0.45 (EtOAc/PE, 1∶5); ^1^H-NMR (500 MHz, CDCl_3_): δ = 8.48 (d, *J* = 2.1 Hz, 1H), 8.42 (dd, *J* = 1.5, 4.8 Hz, 1H), 7.53 (dt, *J* = 1.9, 7.8 Hz, 1H), 7.20 (dd, *J* = 4.8, 7.8 Hz, 1H), 2.57 (m, 1H), 1.23–1.90 ppm (m, 16 H). ^13^C-NMR (125 MHz, CDCl_3_): δ = 149.0, 147.3, 142.8, 134.0, 123.2, 42.5, 36.4, 35.2, 33.5, 32.5, 32.2, 28.2, 27.0, 25.8, 20.9 ppm; IR: ν∼ = 2920, 2856, 1423, 1024, 802, 714, 622 cm^–1^; MS (ESI) *m/z* = 216 [M+H]^+^; HRMS: *m/z* [M+H]^+^ calcd for C_15_H_21_N: 216.1752, found: 216.1744.

#### 5-Pyridin-3-yl-3,3a,4,6a-tetrahydro-1H-pentalen-2-one (2)

The title compound was synthesized according to Method B using **2a** (290 mg, 1.07 mmol ), pyridine-3-boronic acid (170 mg, 1.39 mmol), sodium carbonate (567 mg, 5.35 mmol) and tetrakis- (triphenylphosphine)palladium (0) (61 mg, 0.05 mmol) in dimethoxyethanol (9 mL) and water (3 mL) to give a pale yellow oil (180 mg, 84%) after purification by flash chromatography (EtOAc/n-hexane, 1∶1): *R*_f_ = 0.19 (EtOAc/PE, 2∶1); ^1^H-NMR (500 MHz, CDCl_3_): δ = 8.67 (d, *J* = 1.9 Hz, 1H), 8.48 (dd, *J* = 0.9, 4.7 Hz, 1H), 7.68 (dt, *J* = 1.9, 7.9 Hz, 1H), 7.24 (dd, *J* = 4.7, 7.9 Hz, 1H), 6.14 (m, 1H), 3.63 (m, 1H), 3.09–3.19 (m, 2H), 2.51–2.64 (m, 3H), 2.34 (d, *J* = 18.8 Hz, 1H), 2.09 ppm (dd, *J* = 7.4, 19.0 Hz, 1H); ^13^C-NMR (125 MHz, CDCl_3_): δ = 219.0, 148.6, 147.3, 139.3, 132.8, 131.4, 130.6, 123.2, 46.9, 44.9, 42.6, 40.3, 37.3 ppm; IR: ν∼ = 2904, 2360, 1733, 1567, 1486, 1424, 1411, 1324, 1158, 1119, 1023, 871, 813, 789, 705, 620 cm^–1^; MS (ESI) *m/z* = 200 [M+H]^+^.

#### 5-Pyridin-3-yl-hexahydro-pentalen-2-one (3)

The title compound was synthesized according to Method C using **2** (100 mg, 0.50 mmol) and 5% Pd/C (15% wt) in methanol (10 mL) to give a colorless oil (82 mg, 82%): *R*_f_ = 0.14 (EtOAc/PE, 2∶1); ^1^H-NMR (500 MHz, CDCl_3_): δ = 8.48 (s, 1H), 8.44 (d, *J* = 3.4 Hz, 1H), 7.52 (dt, *J* = 1.7, 7.9 Hz, 1H), 7.22 (dd, *J* = 4.8, 7.8 Hz, 1H), 3.13–3.20 (sept, *J* = 6.8 Hz, 1 H), 2.82–2.91 (m, 2H), 2.55–2.61 (m, 2H), 2.51–2.46 (m, 2H), 2.12–2.17 (dd, *J* = 4.2, 19.3 Hz, 2H), 1.44–1.50 ppm (m, 2H); ^13^C-NMR (125 MHz, CDCl_3_): δ = 148.9, 147.7, 139.1, 134.0, 123.4, 44.6, 44.2, 41.9, 39.2 ppm; IR: ν∼ = 2949, 1733, 1575, 1427, 1169, 1025, 805, 716 cm^–1^; MS (ESI) *m/z* = 202 [M+H]^+^; HRMS: *m/z* [M+H]^+^ calcd for C_13_H_16_NO: 202.1232, found: 202.1217.

#### 3-(5-Methylidene-1,3a,4,5,6,6a-hexahydro-pentalen-2-yl)-pyridine (4)

To a suspension of methyltriphenylphosphonium bromide (897 mg, 2.51 mmol) in THF (10 mL) was added a solution of n-BuLi in hexane (2.5 M, 1 mL, 2.50 mmol) at –78°C under N_2_. Subsequently, the reaction was stirred at room temperature for 3 h followed by cooling the reaction mixture to –78°C. A solution of **2** (100 mg, 0.50 mmol) in THF (2 mL) was added slowly. The reaction was allowed to be warmed to room temperature and stirred for 24 h. A saturated aqueous solution of NH_4_Cl (3 mL) was added to quench and the resulting mixture was extracted with ethyl acetate (3×15 mL). The combined organic layers were washed with brine (2×20 mL), dried over MgSO_4_ and concentrated in vacuo. The residue was purified by flash chromatography (AcOEt/n-hexane, 1∶10) to give **4** as a colorless oil (72 mg, 73%): *R*_f = _0.44 (EtOAc/PE, 1∶1); ^1^H-NMR (500 MHz, CDCl_3_): δ = 8.66 (s, 1H), 8.44 (s, 1H), 7.66 (dt, *J* = 1.8, 7.8 Hz, 1H), 7.21 (dd, *J* = 4.8, 7.8 Hz, 1H), 6.09 (q, *J* = 2.1 Hz, 1H), 4.79 (q, *J* = 1.8 Hz, 1H), 4.77 (q, *J* = 1.8 Hz, 1H), 3.42 (m, 1H), 2.90–3.00 (m, 2H), 2.54–2.65 (m, 2H), 2.49 (m, 1H), 2.25 (m, 1H), 2.10 ppm (m, 1H); ^13^C-NMR (125 MHz, CDCl_3_): δ = 152.2, 148.0, 147.3, 138.1, 132.7, 132.0, 131.8, 123.1, 105.6, 50.7, 41.4, 40.5, 40.4, 38.2 ppm; IR: ν∼ = 2940, 2843, 1566, 1474, 1411, 1311, 1186, 1023, 880, 796, 706, 620 cm^–1^; MS (ESI) *m/z* = 198 [M+H]^+^; HRMS: *m/z* [M+H]^+^ calcd for C_14_H_16_N: 198.1283, found: 198.1274.

#### 5-Pyridin-3-yl-1,2,3,3a,4,6a-hexahydro-pentalen-2-ol (5)

The title compound was synthesized according to Method D using **2** (50 mg, 0.25 mmol), sodium borohydride (19 mg, 0.50 mmol) in MeOH (5 mL) to give a pale yellow crystal (32 mg, 64%) after purification by flash chromatography (MeOH/CH_2_Cl_2_, 1∶30). *R*_f_ = 0.18 (MeOH/CH_2_Cl_2_, 1∶20); mp: 110–112°C; ^1^H-NMR (500 MHz, CDCl_3_): δ = 8.37 (d, *J* = 1.6 Hz, 1H), 8.44 (dd, *J* = 1.1, 4.7 Hz, 1H), 7.68 (dt, *J* = 1.9, 7.9 Hz, 1H), 7.22 (ddd, *J* = 0.4, 4.7, 7.9 Hz, 1 H), 6.24 (q, *J* = 2.1 Hz, 1H), 4.25 (quin, *J* = 5.7 Hz, 1H), 3.32–3.38 (m, 1H), 3.01–3.07 (ddt, *J* = 2.1, 9.3, 16.2 Hz, 1H), 2.83–2.90 (tquin, *J* = 2.4, 9.0 Hz, 1H), 2.62–2.67 (dq, *J* = 2.4, 16.2 Hz, 1H), 2.23 (m, 1H), 2.14 (m, 1H), 1.75 (s, br, 1H), 1.52–1.62 ppm (m, 2H); ^13^C-NMR (125 MHz, CDCl_3_): δ = 148.1, 147.3, 137.3, 132.8, 132.4, 132.0, 123.2, 74.6, 49.7, 43.9, 40.7, 38.5 ppm; IR: ν∼ = 3221, 2957, 2929, 1444,, 1344, 1088, 1041, 810, 711, 629 cm^–1^; MS (ESI) *m/z* = 202 [M+H]^+^; HRMS: *m/z* [M+H]^+^ calcd for C_13_H_16_NO: 202.1232, found: 202.1223.

#### 5-Pyridin-3-yl-octahydro-pentalen-2-ol (6)

The title compound was synthesized according to Method D using **3** (40 mg, 0.22 mmol), sodium borohydride (15 mg, 0.40 mmol) in MeOH (5 mL) to give a pale yellow semisolid (30 mg, 74%) after purification by flash chromatography (MeOH/CH_2_Cl_2_, 1∶30). *R*_f_ = 0.17 (MeOH/CH_2_Cl_2_, 1∶20); ^1^H-NMR (500 MHz, CDCl_3_): δ = 8.47 (d, *J* = 2.3 Hz, 1H), 8.39 (dd, *J* = 1.6, 4.8 Hz, 1 H), 7.55 (dt, *J* = 1.8, 7.9 Hz, 1H), 7.18 (dd, *J* = 4.8, 7.9 Hz, 1H), 4.38 (quin, *J* = 5.6 Hz, 1 H), 2.97–3.04 (sept, *J* = 6.1 Hz, 1H), 2.53–2.75 (m, 2 H), 2.27–2.35 (m, 2H), 2.18 (s, br, 1H), 2.03–2.09 (m, 2H), 1.61–1.68 (m, 2H), 1.52–1.56 ppm (m, 2H); ^13^C-NMR (125 MHz, CDCl_3_): δ = 148.9, 147.2, 139.8, 134.3, 123.2, 76.5, 45.9, 42.7, 42.0 ppm; IR: ν∼ = 3318, 2936, 1578, 1426, 1097, 1027, 983, 713, 625 cm^–1^; MS (ESI) *m/z* = 204 [M+H]^+^; HRMS: *m/z* [M+H]^+^ calcd for C_13_H_18_NO: 204.1388, found: 204.1379.

#### 5-Pyridin-3-yl-cyclooct-4-enone (7)

The title compound was synthesized according to Method B using **7a** (103 mg, 0.38 mmol), pyridine-3-boronic acid (60 mg, 0.49 mmol), sodium carbonate (200 mg, 1.89 mmol) and tetrakis(triphenylphosphine) palladium (0) (22 mg, 0.02 mmol) in dimethoxyethanol (6 mL) and water (2 mL) to give a pale yellow oil (50 mg, 65%) after purification by flash chromatography (EtOAc/n-hexane, 1∶5). *R*_f_ = 0.21 (EtOAc/PE, 2∶1); ^1^H-NMR (500 MHz, CDCl_3_): δ = 8.58 (d, *J* = 1. 7 Hz, 1H), 8.46 (d, *J* = 4.7 Hz, 1H), 7.60 (dt, *J* = 1.7, 7.9 Hz, 1H), 7.21 (dd, *J* = 4.7, 7.9 Hz, 1H), 6.06 (m, 1H), 2.60–2.65 (m, 6H), 2.51 (m, 2H), 1.71–1.76 ppm (m, 2H); ^13^C-NMR (125 MHz, CDCl_3_): δ = 212.3, 146.7, 145.9, 137.2, 136.5, 131.7, 127.4, 121.6, 45.6, 38.7, 28.3, 21.9, 21.8 ppm; IR: ν∼ = 2934, 1703, 1471, 1412, 1337, 1110, 892, 803, 712, 618 cm^–1^; MS (ESI) *m/z* = 202 [M+H]^+^; HRMS: *m/z* [M+H]^+^ calcd for C_13_H_16_NO: 202.1232, found: 202.1224.

#### 5-Pyridin-3-yl-cyclooctanone (8)

The title compound was synthesized according to Method C using **7** (87 mg, 0.43 mmol) and 5% Pd/C (13 mg) in methanol (10 mL) to give a colorless oil (60 mg, 69%) after purification by flash chromatography (EtOAc/n-hexane, 1∶2). *R*_f_ = 0.21 (EtOAc/PE, 2∶1); ^1^H-NMR (500 MHz, CDCl_3_): δ = 8.40 (d, *J* = 4.7 Hz, 1H); 8.37 (s, 1H), 7.40 (dt, *J* = 1.9, 7.9 Hz, 1H), 7.17 (dd, *J* = 4.7, 7.9 Hz, 1H), 2.64–2.69 (m, 2H), 2.34–2.40 (m, 3H), 2.13–2.21 (m, 2H), 1.81–1.90 ppm (m, 6H); ^13^C-NMR (125 MHz, CDCl_3_): δ = 217.1, 148.5, 147.4, 144.6, 134.0, 123.4, 42.0, 40.7, 36.1, 25.2 ppm; IR: ν∼ = 2930, 2858, 1696, 1446, 1258, 1209, 1024, 979, 849, 716 cm^–1^; MS (ESI) *m/z* = 204 [M+H]^+^; HRMS: *m/z* [M+H]^+^ calcd for C_13_H_18_NO: 204.1388, found: 204.1380.

#### 5-Pyridin-3-yl-cyclooct-4-enol (9)

The title compound was synthesized according to Method D using **7** (60 mg, 0.30 mmol), sodium borohydride (23 mg, 0.60 mmol) in MeOH (5 mL) to give a light yellow oil (30 mg, 49%) after flash chromatography (MeOH/CH_2_Cl_2_, 1∶30). *R*_f_ = 0.14 (MeOH/CH_2_Cl_2_, 1∶20); ^1^H-NMR (500 MHz, CDCl_3_): δ = 8.61 (d, *J* = 2.2 Hz, 1H), 8.43 (dd, *J* = 1.5, 4.8 Hz, 1H), 7.64 (dt, *J* = 2.2, 7.9 Hz, 1H), 7.21 (dd, *J* = 4.8, 7.9 Hz, 1H), 6.08 (t, *J* = 8.2 Hz, 1H), 3.81 (m, 1H), 2.60 (t, *J* = 6.3 Hz, 2H), 2.41–2.49 (m, 1H), 2.24–2.30 (m, 1H), 1.98–2.04 (m, 1H), 1.92 (br, 1H), 1.82–1.87 (m, 1H), 1.74–1.79 (m, 2H), 1.59–1.70 ppm (m, 2H); ^13^C-NMR (125 MHz, CDCl_3_): δ = 147.7, 147.1, 138.1, 137.0, 132.9, 129.3, 123.1, 72.8, 38.3, 36.2, 28.6, 24.9, 24.2 ppm; IR: ν∼ = 3329, 2924, 2853, 1472, 1414, 1077, 1041, 1023, 965, 803, 709 cm^–1^; MS (ESI) *m/z* = 204 [M+H]^+^; HRMS: *m/z* [M+H]^+^ calcd for C_13_H_18_NO: 204.1388, found: 204.1380.

#### (endo/exo)-5-Pyridin-3-yl-cyclooctanol (10)

The title compound was synthesized according to Method D using **8** (96 mg, 0.47 mmol), sodium borohydride (36 mg, 0.95 mmol) in MeOH (5 mL) to give a colorless oil (72 mg, 75%) after flash chromatography (MeOH/CH_2_Cl_2_, 1∶30). *R*_f_ = 0.24 (MeOH/CH_2_Cl_2_, 1∶20); ^1^H-NMR (500 MHz, CDCl_3_): δ = 8.41 (m, 2H), 7.46 (m, 1H), 7.19 (m, 1H), 3.86–4.04 (m, 1H), 2.66–2.83 (m, 1H), 1.90–2.01 (m, 4H), 1.79–1.87 (m, 3H), 1.69–1.77 ppm (m, 6H); ^13^C-NMR (125 MHz, CDCl_3_): δ = 148.7, 147.1, 144.9, 134.1, 123.4, 71.7, 71.6, 41.6, 40.3, 35.6, 35.2, 34.3, 22.6, 21.7 ppm; IR: ν∼ = 3362, 2923, 1577, 1425, 1053, 990, 796, 715, 624 cm^–1^; MS (ESI) *m/z* = 206 [M+H]^+^; HRMS: *m/z* [M+H]^+^ calcd for C_13_H_20_NO: 206.1545, found: 206.1536.

#### 3-Cyclooct-1-enyl-pyridine (11)

The title compound was synthesized according to Method B using **11a** (300 mg, 1.16 mmol), pyridine-3-boronic acid (186 mg, 1.51 mmol), sodium carbonate (614 mg, 5.81 mmol) and tetrakis(triphenylphosphine)palladium (0) (133 mg, 0.11 mmol) in dimethoxyethanol (12 mL) and water (4 mL) to give a pale yellow oil (153 mg, 71%) after purification by flash chromatography (EtOAc/n-hexane, 1∶5). *R*_f_ = 0.38 (EtOAc/PE, 1∶5); ^1^H-NMR (500 MHz, CDCl_3_): δ = 8.65 (d, *J* = 2.1 Hz, 1H), 8.43 (dd, *J* = 1.3, 4.7 Hz, 1H), 7.66 (dt, *J* = 2.1, 7.9 Hz, 1H), 7.20 (dd, *J* = 4.7, 7.9 Hz, 1H), 6.04 (t, *J* = 8.3 Hz, 1H), 2.61 (t, *J* = 6.3 Hz, 2H), 2.30 (m, 2H), 1.57–1.66 (m, 4H),1.50–1.56 ppm (m, 4H); ^13^C-NMR (125 MHz, CDCl_3_): δ = 147.6, 147.4, 138.4, 137.4, 132.8, 129.8, 123.0, 29.8, 29.2, 28.2, 27.4, 26.8, 26.0 ppm; IR: ν∼ = 2922, 2850, 1471, 1412, 1021, 802, 709, 626 cm^–1^; MS (ESI) *m/z* = 188 [M+H]^+^; HRMS: *m/z* [M+H]^+^ calcd for C_13_H_18_N: 188.1439, found: 188.1430.

#### 3-Cyclooctyl-pyridine (12)

The title compound was synthesized according to Method C using **11** (160 mg, 0.85 mmol) and 5% Pd/C (16 mg) in methanol (15 mL) to give a colorless oil (150 mg, 93%) after purification by flash chromatography (EtOAc/n-hexane, 1∶10). *R*_f_ = 0.22 (EtOAc/PE, 1∶5); ^1^H-NMR (500 MHz, CDCl_3_): δ = 8.45 (d, *J* = 1.8 Hz, 1H), 8.40 (dd, 1H, *J* = 1.5, 4.8 Hz, 1H), 7.48 (dt, *J* = 4.8, 7.8 Hz, 1H), 7.18 (dd, *J* = 4.8, 7.8 Hz, 1H), 2.81–2.76 (m, 1H), 1.73–1.86 (m, 6H), 1.58–1.68 ppm (m, 8H); ^13^C-NMR (125 MHz, CDCl3): δ = 149.0, 147.0, 145.1, 134.2, 123.3, 42.1, 34.2, 26.8, 26.2, 25.8 ppm; IR: ν∼ = 2920, 2851, 1574, 1476, 1446, 1423, 793, 715, 640, 613 cm^–1^; MS (ESI) *m/z* = 190 [M+H]^+^; HRMS: *m/z* [M+H]^+^ calcd for C_13_H_20_N: 190.1596, found: 190.1587.

#### 3-Cyclodec-1-enyl-pyridine (13)

The title compound was synthesized according to Method B using **13a** (410 mg, 1.43 mmol), pyridine-3-boronic acid (228 mg, 1.86 mmol), sodium carbonate (758 mg, 7.15 mmol) and tetrakis (triphenylphosphine)palladium (0) (83 mg, 0. 07 mmol) in dimethoxyethanol (15 mL) and water (5 mL) to give a pale yellow oil (205 mg, 67%) after purification by flash chromatography (EtOAc/n-hexane, 1∶20). *R*_f_ = 0.19 (EtOAc/PE,1∶10); ^1^H-NMR (500 MHz, CDCl_3_): δ = 8.64 (d, *J* = 1.9 Hz, 1H), 8.46 (dd, *J* = 1.4, 4.7 Hz, 1H), 7.64 (dt, *J* = 1.9, 7.9 Hz, 1H), 7.21 (dd, *J* = 4.7, 7.9 Hz, 1H), 5.70 (t, *J* = 8.4 Hz, 1H), 2.71 (t, *J* = 6.1 Hz, 2H), 2.43 (q, *J* = 6.7 Hz, 2H), 1.67 (m, 2H), 1.47–1.40 (m, 8H), 1.22 ppm (m, 2H); ^13^C-NMR (125 MHz, CDCl_3_): δ = 148.1, 147.8, 138.3, 136.8, 133.6, 131.5, 123.0, 27.6, 26.9, 26.6, 26.1, 25.7, 24.7, 21.2, 20.4 ppm; IR: ν∼ = 2921, 2850, 1474, 1444, 1411, 1022, 885, 804, 757, 712, 625 cm^–1^; MS (ESI) *m/z* = 216 [M+H]^+^; HRMS: *m/z* [M+H]^+^ calcd for C_15_H_22_N: 216.1752, found: 216.1744.

#### 3-Cyclodecyl-pyridine (14)

The title compound was synthesized according to Method C using **13** (63 mg, 0.29 mmol) and 5% Pd/C (10 mg) in methanol (6 mL) to give a colorless oil (48 mg, 75%) after purification by flash chromatography (EtOAc/n-hexane, 1∶10). *R*_f_ = 0.14 (EtOAc/PE, 1∶5); ^1^H-NMR (500 MHz, CDCl_3_): δ = 8.45 (d, *J* = 2.0 Hz, 1H), 8.40 (dd, *J* = 1.6, 4.8 Hz, 1H), 7.50 (dt, *J* = 2.0, 7.9 Hz, 1H), 7.19 (dd, *J* = 4.8, 7.9 Hz, 1H), 2.97 (m, 1H),1.88–1.94 (m, 2H), 1.69–1.45 ppm (m, 16H); ^13^C-NMR (125 MHz, CDCl_3_): δ = 149.4, 147.2, 143.8, 134.6, 123.3, 39.6, 32.5, 25.1, 25.0, 23.9 ppm; IR: ν∼ = 2923, 2869, 1574, 1479, 1443, 1423, 1024, 806, 715, 632, 615 cm^–1^; MS (ESI) *m/z* = 218 [M+H]^+^; HRMS: *m/z* [M+H]^+^ calcd for C_15_H_24_N: 218.1909, found: 218.1900.

#### 3-Cyclododec-1-enyl-pyridine (15)

The title compound was synthesized according to Method B using **15a** (300 mg, 0.95 mmol), pyridine-3-boronic acid (153 mg, 1.24 mmol), sodium carbonate (506 mg, 4.77 mmol) and tetrakis(triphenylphosphine) palladium (0) (110 mg, 0. 09 mmol) in dimethoxyethanol (12 mL) and water (4 mL) to give a pale yellow oil (140 mg, 60%) after purification by flash chromatography (EtOAc/n-hexane, 1∶5). *R*_f_ = 0.38 (EtOAc/PE, 1∶5); ^1^H-NMR (500 MHz, CDCl_3_): δ = 8.54 (d, *J* = 2.1 Hz, 1H), 8.48 (dd, *J* = 1.6, 4.8 Hz, 1H), 7.56 (dt, *J* = 2.1, 7.9 Hz, 1H), 7.26 (dd, *J* = 4.8, 7.9 Hz, 1H), 5.86 (t, *J* = 8.1 Hz, 1H), 2.46 (t, *J* = 5.8 Hz, 2H), 2.00 (m, 2H),1.28–1.58 ppm (m, 16H); ^13^C-NMR (125 MHz, CDCl_3_): δ = 149.3, 147.4, 136.0, 135.5, 134.7, 132.1, 122.8, 36.5, 28.8, 27.3, 26.9, 25.9, 25.8, 24.0, 23.7, 23.5, 23.0 ppm; IR: ν∼ = 2925, 2855, 1461, 1408, 1022, 814, 716, 622 cm^–1^; MS (ESI) *m/z* = 244 [M+H]^+^; HRMS: *m/z* [M+H]^+^ calcd for C_17_H_26_N: 244.2065, found: 244.2057.

#### 3-Cyclododecyl-pyridine (16)

The title compound was synthesized according to Method C using **15** (30 mg, 0.22 mmol) and 5% Pd/C (10 mg) in methanol (6 mL) to give a colorless oil (26 mg, 87%) after purification by flash chromatography (EtOAc/n-hexane, 1∶10). *R*_f_ = 0.22 (EtOAc/PE, 1∶5); ^1^H-NMR (500 MHz, CDCl_3_): δ = 8.43 (s, 1H),8.41 (d, *J* = 4.7 Hz, 1H),7.48 (d, *J* = 7.8, 1H), 7.19 (dd, *J* = 4.7, 7.8 Hz, 1H), 2.76 (quin, *J* = 6.3 Hz, 1H), 1.79–1.85 (m, 2H), 1.26–1.50 ppm (m, 20H); ^13^C-NMR (125 MHz, CDCl_3_): δ = 149.6, 147.2, 142.5, 134.7, 123.2, 37.3, 31.2, 23.8, 23.7, 23.3, 23.2, 22.5 ppm; IR: ν∼ = 2929, 2848, 1575, 1469, 1424, 1026, 812, 733, 716, 609 cm^–1^; MS (ESI) *m/z* = 246 [M+H]^+^; HRMS: *m/z* [M+H]^+^ calcd for C_17_H_28_N: 246.2222, found: 246.2213.

#### (±)-8a-Methyl-6-pyridin-3-yl-3,7,8,8a-tetrahydro-2H-naphthalen-1-one (17)

The title compound was synthesized according to Method B using **17a** (290 mg, 0.93 mmol), pyridine-3-boronic acid (149 mg, 1.21 mmol), sodium carbonate (492 mg, 4.65 mmol) and tetrakis(triphenylphosphine)palladium (0) (54 mg, 0.05 mmol) in dimethoxyethanol (12 mL) and water (4 mL) to give a pale yellow oil (200 mg, 90%) after purification by flash chromatography (EtOAc/n-hexane, 1∶2). *R*_f_ = 0.24 (EtOAc/PE, 1∶1); ^1^H-NMR (500 MHz, CDCl_3_): δ = 8.72 (d, *J* = 1.8 Hz, 1H), 8.46 (dd, *J* = 1.0, 4.7 Hz, 1H), 7.72 (dt, *J* = 1.8, 8.0 Hz, 1H), 7.24 (dd, *J* = 4.7, 8.0 Hz, 1H), 6.54 (s, 1H), 5.86 (t, *J* = 4.4 Hz, 1H), 2.80 (ddd, *J* = 5.7, 7.2, 15.6 Hz, 1H), 2.66–2.73 (m, 1H), 2.50–2.62 (m, 3H), 2.38–2.44 (m, 1H), 2.12 (ddd, *J* = 2.2, 4.6, 13.4 Hz, 1H), 1.63–1.69 (m, 1H), 1.25 ppm (s, 3H); ^13^C-NMR (125 MHz, CDCl_3_): δ = 214.6, 148.2, 146.6, 139.8, 136.1, 132.5, 132.1, 125.7, 125.0, 123.1, 44.8, 35.4, 28.8, 24.3, 23.7, 22.6 ppm; IR: ν∼ = 2925, 2358, 1706, 1446, 1414, 1094, 1045, 896, 807, 707 cm^–1^; MS (ESI) *m/z* = 240 [M+H]^+^.

#### 3-Bicyclo[2.2.1]hept-2-en-2-yl-pyridine (18)

The title compound was synthesized according to Method B using **18a** (410 mg, 1.69 mmol), pyridine-3-boronic acid (270 mg, 2.20 mmol), sodium carbonate (896 mg, 8.45 mmol) and tetrakis(triphenylphosphine)-palladium (0) (98 mg, 0.08 mmol) in dimethoxyethanol (18 mL) and water (6 mL) to give a pale yellow oil (237 mg, 82%) after purification by flash chromatography (EtOAc/n-hexane, 1∶10). *R*_f = _0.21 (EtOAc/PE, 1∶5); ^1^H-NMR (500 MHz, CDCl_3_): δ = 8.66 (d, *J* = 2.1 Hz, 1H), 8.41 (d, *J* = 4.7 Hz, 1H), 7.65 (dt, *J* = 2.1, 7.9 Hz, 1H), 7.20 (dd, *J* = 4.7, 7.9 Hz, 1H), 6.39 (d, *J* = 2.5 Hz, 1H), 3.32 (s, 1H), 3.02 (s, 1H), 1.76–1.83 (m, 2H), 1.54 (dt, *J* = 1.9, 8.2 Hz, 1H), 1.27 (d, *J* = 8.2 Hz, 1H), 1.09–1.18 ppm (m, 2H); ^13^C-NMR (125 MHz, CDCl_3_): δ = 146.7, 146.6, 144.8, 131.8, 131.8, 131.4, 123.3, 48.0, 43.2, 43.0, 26.5, 24.7 ppm; IR: ν∼ = 2962, 2871, 1684, 1585, 1418, 1301, 1024, 873, 798, 707 cm^–1^; MS (ESI) *m/z* = 172 [M+H]^+^.

#### (endo/exo)-3-Bicyclo[2.2.1]hept-2-yl-pyridine (19)

The title compound was synthesized according to Method C using 18 (190 mg, 0.22 mmol) and 5% Pd/C (30 mg) in methanol (10 mL) to give a colorless oil (171 mg, 90%) after purification by flash chromatography (EtOAc/n-hexane, 1∶10). *R*_f_ = 0.22 (EtOAc/PE, 1∶5); ^1^H-NMR (500 MHz, CDCl_3_): δ = 8.46 (d, *J* = 2.2 Hz, 1H), 8.40 (dd, *J* = 1.4, 4.7 Hz, 1H), 7.49 (d, *J* = 7.8 Hz, 1H ), 7.20 (dd, *J* = 4.7, 7.8 Hz, 1H), 3.20 (m, 1H), 2.42 (t, *J* = 3.9 Hz, 1H), 2.35 (t, *J* = 4.3 Hz, 1H), 1.97–2.04 (tt, *J* = 3.9, 12.1 Hz,1H), 1.53–1.60 (m, 2H), 1.46 (m, 1H), 1.40 (ddd, *J* = 2.3, 5.7, 12.5 Hz, 1H), 1.28–1.34 (m, 1H), 1.18–1.24 ppm (m, 2H); ^13^C-NMR (125 MHz, CDCl_3_): δ = 150.1, 146.9, 138.8, 135.4, 122.9, 43.6, 42.2, 40.5, 37.4, 34.0, 30.0, 22.7 ppm; IR: *ν∼* = 2951, 2872, 1478, 1420, 1025, 802, 714, 615 cm^–1^; MS (ESI) *m/z* = 174 [M+H]^+^; HRMS: *m*/*z* [M+H]^+^ calcd for C_12_H_16_N: 174.1283, found: 174.1273.

#### 8-Methyl-3-pyridin-3-yl-8-aza-bicyclo[3.2.1]oct-2-ene (20)

The title compound was synthesized according to Method B using 18a (391 mg, 1.44 mmol), pyridine-3-boronic acid (230 mg, 1.87 mmol), sodium carbonate (763 mg, 7.2 mmol) and tetrakis(triphenylphosphine)palladium (0) (83 mg, 0.07 mmol) in dimethoxyethanol (18 mL) and water (6 mL) to give a pale yellow oil (201 mg, 70%) after purification by flash chromatography (EtOAc/n-hexane, 1∶10). *R*_f_ = 0.30 (CHCl_2_/MeOH/Et_3_N, 100∶10∶1); ^1^H-NMR (500 MHz, CDCl_3_): δ = 8.63 (d, *J = *1.9 Hz, 1H), 8.48 (dd, *J* = 1.3, 4.7 Hz, 1H), 7.64 (dt, *J* = 1.9, 7.9 Hz, 1H), 7.24 (dd, *J* = 4.7, 7.9 Hz, 1H), 6.32 (dt, *J* = 1.5, 5.6 Hz, 1H), 3.66 (tt, *J* = 5.8, 17.6 Hz, 2H), 2.98 (dd, *J* = 3.9, 17.6 Hz, 1H), 2.55 (s, 3H), 2.28–2.35 (m, 1H), 2.18–2.25 (m, 2H), 2.02 (m, 1H), 1.72 ppm (m, 1H); ^13^C-NMR (125 MHz, CDCl_3_): δ = 148.6, 146.4, 134.6, 132.1, 130.5, 127.1, 123.2, 59.8, 58.4, 36.3, 33.1, 29.6, 29.1 ppm; IR: *ν∼* = 2941, 1415, 1260, 1156, 1030, 802, 708, 638 cm^–1^; MS (ESI) *m/z* = 200 [M]^+^.

#### (endo/exo)-8-Methyl-3-pyridin-3-yl-8-aza-bicyclo[3.2.1]octane (21)

The title compound was synthesized according to Method C using 20 (100 mg, 0.50 mmol) and 5% Pd/C (15 mg) in methanol (10 mL) to give a colorless oil (92 mg, 91%) after purification by flash chromatography (CHCl_2_/MeOH/Et_3_N, 100∶10∶1). *R*_f_ = 0.18 (CHCl_2_/MeOH/Et_3_N, 100∶10∶1); ^1^H-NMR (500 MHz, CDCl_3_): δ = 8.55 (d, *J* = 2.0 Hz, 1H), 8.40 (dd, *J* = 1.5, 4.7 Hz, 1H), 7.56 (dt, *J* = 1.7, 7.9 Hz, 1H), 7.18 (dd, *J* = 4.7, 7.9 Hz, 1H), 3.20–3.27 (m, 2H), 3.07 (quin, *J* = 8.0 Hz, 1H), 2.43 (m, 2H), 2.24 (s, 3H), 2.03–2.12 (m, 2H), 1.57 (ddd, *J* = 1.3, 8.4, 13.9 Hz, 2H), 1.43 ppm (m, 2H); ^13^C-NMR (125 MHz, CDCl_3_): δ = 149.2, 147.1, 141.4, 134.5, 123.0, 59.5, 40.6, 38.3, 30.3, 28.1 ppm; IR: *ν∼* = 2935, 1575, 1478, 1451, 1332, 1024, 799, 714, 623 cm^–1^; MS (ESI) *m/z* = 202 [M]^+^.

### Docking Study

Homology model of CYP11B2 was employed as described before [13b], no conformational search of the ligand was performed. The automatic active-site detection was switched on; while heme iron was selected as active-site origin and the radius of active site was set to 19 Å. A distance constraint of 1.9 to 2.5 Å between the sp2 hybrid N and the iron was set as well. Ligand was docked in 50 independent genetic algorithm (GA) iterations for each of the three GOLD-docking runs. Moreover, the goldscore.p450_pdb parameters were exploited and the genetic algorithm default parameters were set.

## Supporting Information

Information S1 The synthetic procedures and characterization of all intermediates, as well as the HPLC purity control of all final compounds.
(DOC)
