# Supplementary material for: 3-Pyridyl Substituted Aliphatic Cycles as CYP11B2 Inhibitors: Aromaticity Abolishment of the Core Significantly Increased Selectivity over CYP1A2
Source: PLoS One. 2012 Nov 1;7(11):e48048. doi: 10.1371/journal.pone.0048048 (PMC3486838; doi:10.1371/journal.pone.0048048)
Supplement: Information S1 — The synthetic procedures and characterization of all intermediates, as well as the HPLC purity control of all final compounds. (DOC) [file pone.0048048.s001.doc]

**Supporting Information**

3-Pyridyl Substituted Aliphatic Cycles as CYP11B2 Inhibitors: Aromaticity Abolishment of the Core Significantly Increased Selectivity over CYP1A2

Lina Yin,^[1,2]^ Qingzhong Hu, ^[1]*^ Rolf W. Hartmann^[1]*^

[1] L. Yin, Q. Hu, Prof. Dr. R. W. Hartmann
Pharmaceutical and Medicinal Chemistry, Saarland University & Helmholtz Institute for Pharmaceutical Research Saarland (HIPS), Campus C2-3, D‑66123 Saarbrücken, Germany
Prof. Dr. R. W. Hartmann: Fax: (+49) 681-302-70308; E-mail: [rolf.hartmann@helmholtz-hzi.de](mailto:rolf.hartmann@helmholtz-hzi.de).; Homepage: <http://www.helmholtz-hzi.de/?id=3897>. Dr. Qingzhong Hu: E-mail: [q.hu@mx.uni-saarland.de](mailto:q.hu@mx.uni-saarland.de).

[2] L. Yin
ElexoPharm GmbH, Campus A1, D-66123 Saarbrücken (Germany)

**Contents**

1. The synthetic procedures and characterization of all intermediates.
2. HPLC purity control of all final compounds.

**Method A: Triflation**

To a solution of 2,6-di-*tert*-butyl-4-methylpyridine (7.23 mmol) and the corresponding ketone (6.57 mmol) in CH_2_Cl_2_ (20 mL) was added trifluoromethanesulfonic anhydride (7.16 mmol) dropwise at 0 °C under nitrogen. A white precipitate was formed soon. The reaction mixture was warmed to room temperature and further stirred for 4 h. The solvent was removed in vacuo, and the resulting residue was diluted with petroleum ether (20 mL). After removal of the solid by filtration, the filtrate was concentrated in vacuo to give a crude product which was used in the next step without further purification.

**Method B: Suzuki coupling**

A suspension of enol triflate (4.56 mmol), pyridine-3-boronic acid (5.92 mmol), sodium carbonate (22. 8 mmol) and tetrakis(triphenylphosphine)palladium (0) (0.23 mmol) in dimethoxyethanol (45 mL) and water (15 mL) was stirred at 90 °C under nitrogen for 2 h. The reaction mixture was cooled to room temperature naturally and diluted with water (10 mL). The aqueous layer was extracted with ethyl acetate (2 x 30 mL) and the combined organic layers were washed with brine twice and dried over MgSO_4_. After evaporation in vacuo, the resulting residue was purified by flash chromatography to afford the corresponding product.

**Trifluoro-methanesulfonic acid 3,4,4a,5,6,7,8,8a-octahydro-naphthalen-2-yl ester (1b):** The title compound was synthesized according to Method A using a mixture of *cis* and *trans* of 2-decalone (1.00 g, 6.57 mmol), trifluoromethanesulfonic anhydride (1.20 mL, 7.16 mmol) and 2, 6-di-*tert*-butyl-4-methylpyridine (1.48 g, 7.23 mmol) in CH_2_Cl_2_ (20 mL) to give a yellow oil (1.30 g).

**A isomer mixture of 3-(3,4,4a,5,6,7,8,8a-octahydro-naphthalen-2-yl)-pyridine and 3-(1,4,4a,5,6,7,8,8a-octahydro-naphthalen-2-yl)-pyridine (1a):** The title compound was synthesized according to Method B using **1b** (1.29 g, 4.54 mmol ), pyridine-3-boronic acid (0.73 g, 5.92 mmol), sodium carbonate (2.41 g, 22.8 mmol) and tetrakis (triphenylphosphine)palladium (0) (0.24 g, 0.21 mmol) in dimethoxyethanol (45 mL) and water (15 mL) to give a pale yellow oil (0.86 mg, 88%) after purification by flash chromatography (EtOAc / n-hexane,1:10): *R*_f_ = 0.45 (EtOAc/PE,1:5); MS (ESI) *m/z* = 214 [*M*+H]^+^.

***cis*-Trifluoro-methanesulfonic acid 5-oxo-1,3a,4,5,6,6a-hexahydro-pentalen-2-yl ester (2a):** The title compound was synthesized according to Method A using *cis*-tetrahydropentalene-2,5(1*H*,3*H*)-dione (300 mg, 2.17 mmol), trifluoromethanesulfonic anhydride (0.36 mL, 2.17 mmol) and 2, 6-di-*tert*-butyl-4-methylpyridine (455 mg, 2.21 mmol) in CH_2_Cl_2_ (15 mL) to give a yellow oil (290 mg).

**Cyclooctane-1,5-diol (7b):** A solution of BH_3_-THF complex in THF (1.0 M, 10 mL, 10.0 mmol) was stirred at 0 °C under N_2_. A solution of 1,5-cyclooctadiene (1.00 g, 9.24 mmol) in THF (2 mL) was added slowly, and the resulting mixture was refluxed for 1 h. Upon cooling to room temperature, to the reaction mixture was added 3 M NaOH (2 mL), followed by 35% H_2_O_2_ (1.7 mL), which was added dropwise at a rate which caused the solution to reflux gently. The resulting mixture was allowed to cool for 30 min and saturated with K_2_CO_3_. After separation, the aqueous layer was extracted with diethyl ether (2 × 8 mL). The combined organic layers were dried over MgSO_4_ and concentrated in vacuo to give a colorless tar. The tar was dissolved in CH_2_Cl_2_ (60 mL), and PCC (9.24 g, 42.9 mmol) was added in three portions. The reaction mixture was refluxed for 2 d, and an additional PCC (4.70 g, 21.8 mmol) was added. After refluxing for 6 h, the reaction was cooled to room temperature and poured to a silica column, which was eluted with 1:1 diethyl ether/n-hexane. An offwhite semisolid (1.07 g, 82%) was obtained. ^1^H-NMR (500 MHz, CDCl_3_): δ = 2.49 (m, 8H), 2.13 ppm (m, 4H); ^13^C-NMR (125 MHz, CDCl_3_): δ = 213.0, 42.2, 22.1 ppm.

**Trifluoro-methanesulfonic acid cyclooct-1-enyl ester (11a):** The title compound was synthesized according to Method A using cyclooctanone (1.00 g, 7.87 mmol), trifluoromethanesulfonic anhydride (1.45 mL, 8.57 mmol) and 2, 6-di-*tert*-butyl-4-methylpyridine (1.78 g, 8.64 mmol) in CH_2_Cl_2_ (20 mL) to give a yellow oil (1.60 g).

**Trifluoro-methanesulfonic acid cyclodec-1-enyl ester (13a):** The title compound was synthesized according to Method A using cyclodecanone (300 mg, 1.94 mmol), trifluoromethanesulfonic anhydride (0.33 mL, 1.94 mmol) and 2, 6-di-*tert*-butyl-4-methylpyridine (407 mg, 1.98 mmol) in CH_2_Cl_2_ (15 mL) to give a yellow oil (410 mg).

**Trifluoro-methanesulfonic acid cyclododec-1-enyl ester (15a):** The title compound was synthesized according to Method A using cyclododecanone (1.00 g, 5.49 mmol), trifluoromethanesulfonic anhydride (1.01 mL, 5.98 mmol) and 2, 6-di-*tert*-butyl-4-methylpyridine (1.24 g, 6.03 mmol) in CH_2_Cl_2_ (20 mL) to give a yellow oil (1.71 g).

**Trifluoro-methanesulfonic acid 4a-methyl-5-oxo-3,4,4a,5,6,7-hexahydro-naphthalen-2-yl ester (17a):** The title compound was synthesized according to Method A using Wieland-Miescher ketone (200 mg, 1.12 mmol), trifluoromethanesulfonic anhydride (0.36 mL, 2.15 mmol) and 2, 6-di-*tert*-butyl-4-methylpyridine (481 mg, 2.34 mmol) in CH_2_Cl_2_ (8 mL) to give a yellow oil (294 mg).

**Trifluoro-methanesulfonic acid bicyclo[2.2.1]hept-2-en-2-yl ester (18a):** The title compound was synthesized according to Method A using norcamphor (500 mg, 4.54 mmol), trifluoromethanesulfonic anhydride (0.83 mL, 4.95 mmol) and 2, 6-di-*tert*-butyl-4-methylpyridine (1.03 g, 5.00 mmol) in CH_2_Cl_2_ (20 mL) to give a yellow oil (410 mg).

**Trifluoro-methanesulfonic acid 8-methyl-8-aza-bicyclo[3.2.1]oct-2-en-3-yl ester (20a):** To a solution of tropinone (500 mg, 4.54 mmol) in THF (20 mL) was added a solution of [lithium hexamethyldisilazide](http://www.made-in-china.com/showroom/cdrongyi/product-detailFMZJgxIPCuko/China-Lithium-Hexamethyldisilazide-CAS-4039-32-1-.html) in THF (1.0 M, 4.3 mL, 4.30 mmol) dropwise at –78 °C. The resulting mixture was stirred at the temperature for 30 min, and then was warmed to 0 °C. A solution of PhNTf_2_ (1.54 g, 4.30 mmol) in THF (2 mL) was added. The reaction was stirred for 1 h at 0 °C, and subsequently stirred overnight at room temperature An aqueous solution of NH_4_Cl (10 mL ) was added to quench and the resulting mixture was separated. The aqueous layer was extracted with ethyl acetate (2 x 20 mL). The combined organic layers were washed with brine (3 x 30 mL), dried over MgSO_4_ and concentrated in vacuo to give a red oil (391 mg), which was directly used for next step.

**HPLC Purity Control of Final Compounds**

The Surveyor®-LC-system consisted of a pump, an autosampler, and a PDA detector. Mass spectrometry was performed on a TSQ® Quantum (Thermo Electron Corporation, Dreieich, Germany). The triple quadrupole mass spectrometer was equipped with an electrospray interface (ESI). The system was operated by the standard software Xcalibur®.

A RP C18 NUCLEODUR® 100-5 (125 × 3 mm) column (Macherey-Nagel GmbH, Duehren, Germany) was used as stationary phase. All solvents were HPLC grade.

In a gradient run the percentage of acetonitrile (containing 0.1 % triflouro-acetic acid) in water was increased from an initial concentration of 3% at 0 min to 100% at 15 min and kept at 100% for 3 min.

The injection volume was 10 µl and flow rate was set to 350 µl/min. MS analysis was carried out at a spray voltage of 3800 V, a capillary temperature of 350 °C and a source CID of 10 V. Spectra were acquired in positive mode from 100 to 1000 m/z and full scan UV-mode. In some cases APC ionization had to be applied.

| **Compd** | **RT (min)** | **Purity [%]** |
| --- | --- | --- |
| **1** | 7.98 | 99.9% |
| **2** | 4.45 | 99.1% |
| **3** | 3.55 | 99.1% |
| **4** | 7.86 | 99.9% |
| **5** | 5.10 | 98.1 % |
| **6** | 5.14 | 99.3 % |
| **7** | 4.46 | 99.0% |
| **8** | 4.56 | 99.0% |
| **9** | 7.72 | 99.9% |
| **10** | 4.61 | 98.7% |
| **11** | 7.81 | 98.4% |
| **12** | 8.07 | 99.9% |
| **13** | 9.01 | 98.0 % |
| **14** | 9.31 | 99.3% |
| **15** | 9.03 | 99.4% |
| **16** | 8.99 | 99.9% |
| **17** | 5.29 | 98.1% |
| **18** | 5.04 | 99.9% |
| **19** | 5.62 | 99.0% |
| **20** | 8.81 | 98.3% |
| **21** | 8.79 | 99.3% |
